# Supplementary material for: Pseudouridine Synthase RsuA Captures an Assembly Intermediate That Is Stabilized by Ribosomal Protein S17
Source: Biomolecules. 2020 May 30;10(6):841. doi: 10.3390/biom10060841 (PMC7356742; doi:10.3390/biom10060841)
Supplement: Supplementary file 1 [file biomolecules-10-00841-s001.pdf]

biomolecules

MDPI

Supplementary Materials

Article

Pseudouridine Synthase RsuA Captures an Assembly Intermediate that Is Stabilized by Ribosomal Protein S17

Kumudie Jayalath, Sean Frisbie, Minhchau To and Sanjaya Abeysirigunawardena *

Department of Chemistry and Biochemistry, Kent State University, Kent, OH 44242, USA; kjayalat@kent.edu (K.J.); sfrisbie@kent.edu (S.F.); mto1@kent.edu (M.T.)

* Correspondence: sabeysir@kent.edu; Tel.: +1-330-672-2667

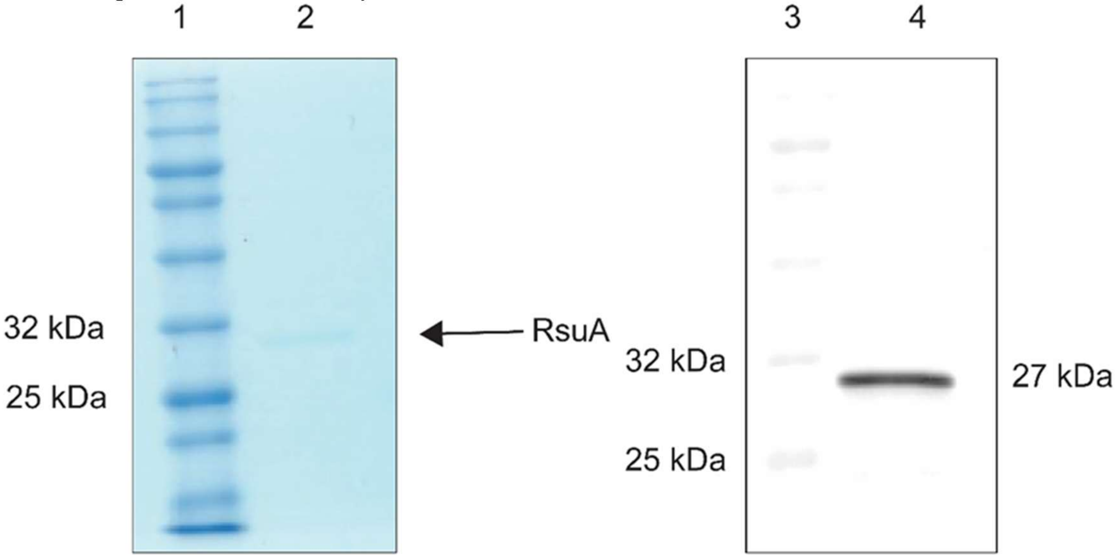

Figure S1. SDS-PAGE of RsuA show single gel band after purification (Lane 2) and fluorescence labeling with Cyanine 5 dye (Lane 4). Lanes 1 and 3 represent protein ladder.

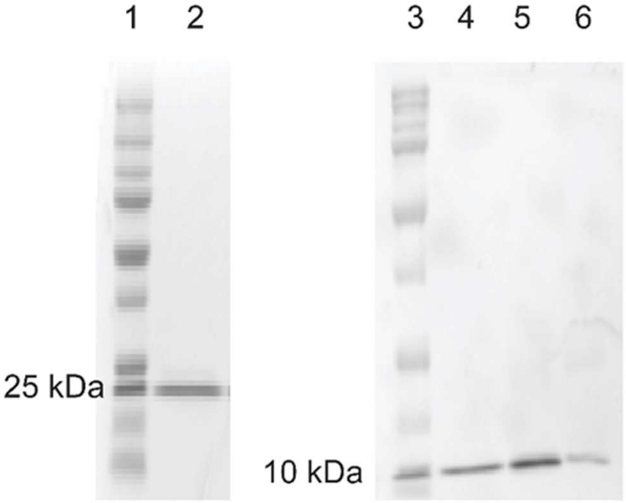

Figure S2. SDS-PAGE gel images for purified ribosomal proteins are shown. Lane 2: Protein S4 (23.1 kDa) Lane 4: Protein S16 (9.1 kDa); Lane 5: Protein S17 (9.5 kDa); Lane 6: protein S20 (9.5 kDa); Lanes 1 and 3: protein ladder.

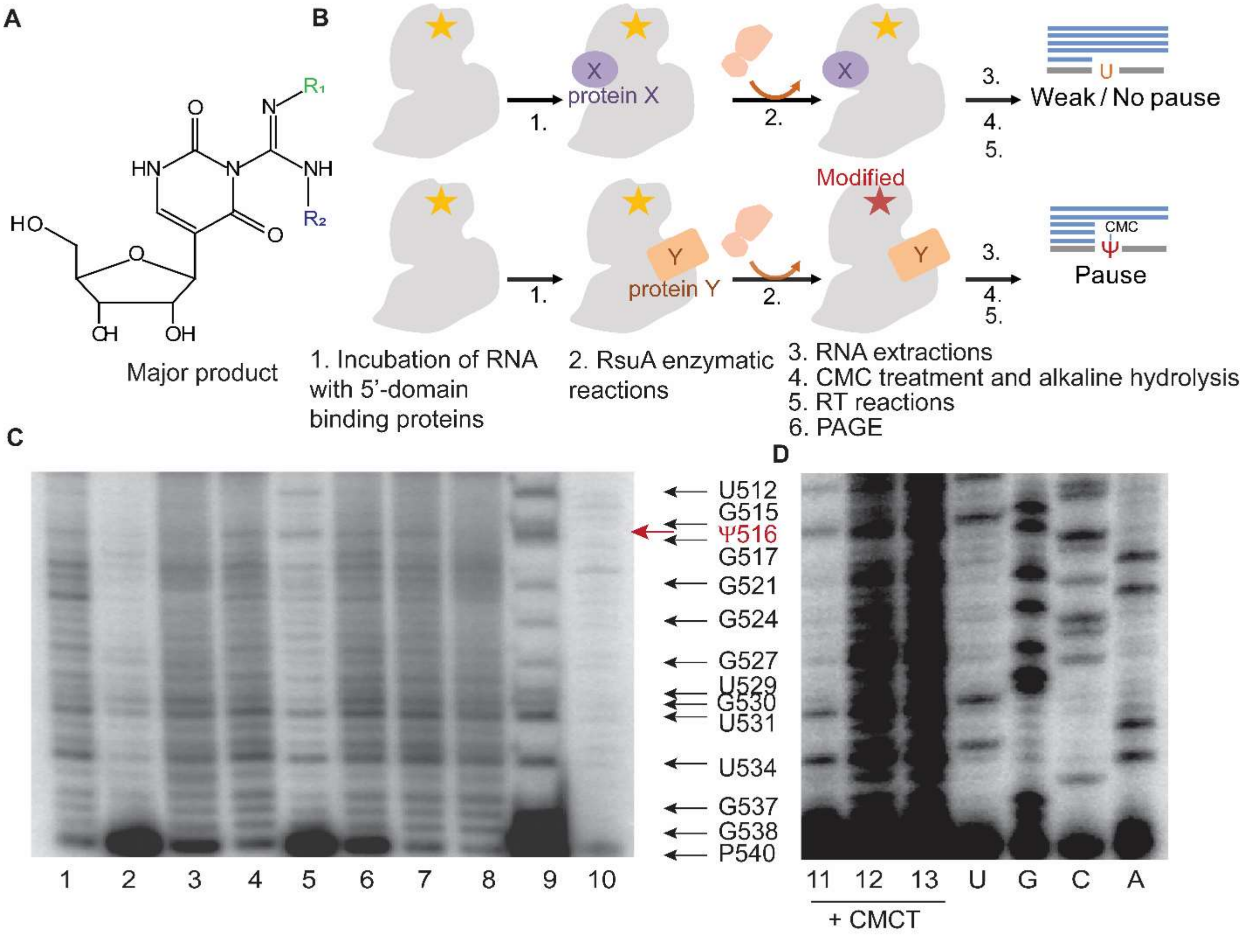

Figure S3. Evaluation of pseudouridylation activity of RsuA. (**A**) Pseudouridine reacts with CMCT and produces CMC adduct shown after alkaline treatment. (**B**) Reverse transcriptase-based assay used in determining pseudouridylase activity of RsuA. (**C**) CMC treated samples show strong pauses at G and U nucleotides. The red arrow indicates the pause at position 516. Lanes of the gel are as follows. Lane 1: RsuA+RNA; Lane 2: RsuA+RNA+S4; Lane 3: RsuA+RNA+S4,S16,S20; Lane 4: RsuA+RNA+S4, S20; Lane 5: RsuA+RNA+S17; Lane 6: RsuA+RNA+S4,S17; Lane 7: RsuA+RNA+S4,S16,S17,S20; Lane 8 +CMCT + Alkaline treated, - RsuA; Lane 9 +CMCT treated, - Alkaline treated, - RsuA; Lane 10: Pause control. Pauses for G and U/Ψ nucleotides determined from CMCT treated sample are indicated by arrows. P540 arrow indicates the unextended primer. Pauses observed below pseudouridylation site may have caused by residual salts and urea leached out from psudouridylation and CMCT reactions (**D**) A gel image for the optimization of CMCT treatment is shown. Lanes 11-13 increasing concentrations of CMCT; U, G, C, A are the sequencing lanes for respective nucleotides.
